# Preliminary results of student survey at the University of Rome “Tor Vergata” practicing sports: a focus on the effects of Dual Career regulation

**DOI:** 10.3389/fspor.2024.1465562

**Published:** 2024-09-20

**Authors:** Ida Cariati, Roberto Bonanni, Manuel Onorati, Virginia Tancredi

**Affiliations:** ^1^ Department of Systems Medicine, “Tor Vergata” University of Rome, Rome, Italy; ^2^ Department of Biomedicine and Prevention, “Tor Vergata” University of Rome, Rome, Italy; ^3^ CUS, “Tor Vergata” University of Rome, Rome, Italy; ^4^ Centre of Space Bio-Medicine, “Tor Vergata” University of Rome, Rome, Italy

**Keywords:** dual career, university, sport, student-athletes, education

## Abstract

The importance of sports and the approval of the Dual Career regulation at the University of Rome “Tor Vergata” are highly significant topics for the well-being and personal development of students. In line with European and international policies, this initiative recognizes the specific needs of student-athletes by offering them tools and flexibility to excel in both sports and academics. However, monitoring student-athletes by Universities requires thorough investigations and the development of initiatives to promote students' adherence to sports practice. Therefore, our study aims to analyze the results of a survey administered to students enrolled at the University of Rome “Tor Vergata” during the academic years 2020/2021, 2021/2022, 2022/2023, and 2023/2024, investigating the effects of the introduction of the Dual Career regulation on the student population. Our preliminary results showed that the introduction of the Dual Career program was associated with a significant increase in the number of students practising sports over the academic years, with greater participation in the academic years 2022/2023 and 2023/2024. Noteworthy, the number of student-athletes who applied to the Dual Career program markedly increased in the last year, with the highest number of enrollments in the macroareas of Medicine and Surgery, Economy, and Engineering. Overall, the Dual Career regulation offers the possibility to plan the study path in a personalized way, dedicated academic support, and flexibility in deadlines, making the University of Rome “Tor Vergata” a model of integration between education and sportiness.

## 1 Introduction

The number of elite athletes engaging in academic-level study has increased in recent years, fostered by the European publication of the Dual Career Guidelines to encourage a balance between sporting life and professional training (1). The process of developing guidelines to ensure the adequacy of Dual Career services for student-athletes became evident as early as 2010 with the study by Aquilina and Henry, who analyzed how European Union (EU) Member States were meeting the educational needs of elite young athletes (2). Particularly, EU Member States' positions on Dual Career policies were characterized, finding four different ways of dealing with them. In countries such as France, Hungary, Luxemburg, Spain, Poland and Portugal, a state-centred arrangement was adopted and supported by legislation; while in Belgium, Denmark, Estonia, Germany, Finland, Latvia, Lithuania and Sweden, the State acted as a facilitator able to promote formal agreements between educational and sporting bodies. In countries such as Greece and the United Kingdom, national federations and sports institutes could act as facilitators or mediators by directly engaging in negotiations with educational institutions on behalf of the individual athlete. Finally, in Austria, Cyprus, Malta, the Czech Republic, the Netherlands, Ireland, Slovenia and Italy, a “laisser faire” approach was adopted where no formal structures were in place and any agreements were largely based on individual negotiations. In the Italian context, Aquilina and Henry's study highlighted a problematic situation in terms of guaranteeing flexibility for athletes interested in pursuing a university education, although some sports federations offered financial support to promote the athlete's education (2). Therefore, the Dual Career Guidelines issued by the EU have made it possible to reduce the inequalities between elite athletes in different national contexts by providing the tools to ensure adherence to educational pathways and social inclusion.

The provision of services and facilities to athletes engaged in study programmes should be a prime objective for universities, as it could offer such individuals the possibility of a longer-lasting sports career and embarking on new career paths at the end of their sporting career (3). This is true because properly balancing life as an athlete with life as a student requires constant commitment and a strong motivational drive, qualities necessary to avoid abandoning either career (4). In fact, practising sport at a competitive level involves training, travel and competitions that require considerable physical and mental commitment on a daily basis (5). In addition, study commitments, which include attending classes, taking exams and spending time on preparation, are all factors that determine the outcome of academic life (6).

This constant challenge on two fronts has a major impact on the athlete's life, as relationships with family, peers, partners, as well as with other students and professors, are influenced by the effects of the dual career (7). The importance of the social context in supporting dual-career athletes was highlighted by Tessitore and colleagues who demonstrated a crucial role of athletes' parents in determining their sporting and academic success (8). Nonetheless, reconciling life as an athlete and as a student is not easy, highlighting the need to develop and adopt strategies to safeguard the athletes' educational and professional paths (9).

In this context, the possibility of taking advantage of a regulation that would allow University-registered athletes to manage their commitments more efficiently, in addition to facilitating the dual career of such individuals, could be a decisive factor in favoring the enrolment of additional athletes or sports students in degree programmes (10). Indeed, many professionals are involved in the world of sport, such as referees and coaches, who are constantly working and who could be encouraged to undertake a dual-career degree. In this regard, Fusco and colleagues showed the results of a survey which revealed that elite coaches are highly motivated to join degree programmes in sports science (11). The inclusion of such figures in the University would be a positive change both for these individuals, who could deepen and renew the numerous knowledge acquired in the field, and for academic institutions, which could increase their visibility and reputation in economic and social terms (12).

Importantly, in countries such as Australia, Canada, New Zealand, Qatar and the United States, dual career programmes are well established and student athletes are an important resource for Universities (13, 14). For this reason, in April 2022, the University of Rome “Tor Vergata” approved the “Dual Career” Program that allows athletes, coaches and referees of national and international interest to combine a university career with a competitive one. In fact, the University of Rome “Tor Vergata”, in collaboration with the University Sports Centre (CUS), offers the student population the opportunity to practise sports at a recreational and competitive level in many disciplines. Specifically, the CUS makes use of the technicians of the degree course in Sport Sciences to guarantee students the opportunity to engage in sports such as football, cycling, basketball, judo and many others, also taking advantage of agreements with external sports facilities to offer comprehensive services. The introduction of the “Dual Career” Program has regulated the status of student-athletes, providing concessions on university fees, compulsory attendance of classes and recognition of university credits, as well as the possibility of suspending studies for a year for important sporting commitments, to reconcile a student-athlete's university career and competitive sporting career. Moreover, since the beneficial effects of competitive and recreational sport are well known and widely documented, the monitoring of students practising sport by Universities could be useful to promote initiatives aimed at encouraging students' adherence to the sport practice. Therefore, we analyzed the results of a survey administered to students enrolled at the University of Rome “Tor Vergata” in the academic years 2020/2021, 2021/2022, 2022/2023 and 2023/2024, to (i) determine the number of students engaged in competitive or recreational sporting activities, and (ii) assess the effects of the introduction of the Dual Career regulation.

## 2 Materials and methods

### 2.1 Participants, instrument and procedure

An up-to-date view of the current situation of the student population at the University of Rome “Tor Vergata” was provided by requesting all students enrolled in the 2020/2021, 2021/2022, 2022/2023 and 2023/2024 academic years to complete a survey. The survey was administered online at the final stage of the enrolment process on the Delphi website to allow for temporal and geographical flexibility, ensuring quick completion and effective retrieval of information.

A total of 113,890 students participated in the survey. Specifically, 17,558 students enrolled in the 2020/2021 academic year, 19,994 students enrolled in the 2021/2022 academic year, 25,310 students enrolled in the 2022/2023 academic year, and 51,028 students enrolled in the 2023/2024 academic year.

The survey was structured in a demographic section and a sports section to collect data on various aspects of students' lives. Specifically, the demographic section included questions on age, the degree course attended and the year of enrolment at University. The sports section consisted of questions on the type of sport played, the level of competition, and the frequency and duration of training. Specifically, based on the responses of the students who participated in the survey, three categories were considered: competitive sport, which includes students who engage in sport by participating in competitions and intensive training; recreational sport, which includes students who participate in sporting activities for recreation, physical and social wellbeing, without the intensity and commitment required at the competitive level; and no sport, i.e., students who do not engage in sporting activities for a variety of reasons, including lack of time, interest, or other priorities. All questions were closed to collect quantitative data that could be easily analyzed, offering space to each student for additional answers. Specifically, the questions in the sports section included:

“Do you practise sport?”, with three answer options: Yes, No, Sometimes

“What is the level at which you practise sport?”, with three answer options: Competitive, Recreational, I do not practise sport

“What type of sport do you practise?”, with four response options: Individual, Couple, Team, Other (specify)

“What sport do you practise?”, with six answer options: Football, Basketball, Swimming, Athletics, Tennis, Other (specify)

“In which time frame do you prefer to practise sport?”, with five answer options: 8–13, 13–16, 16–23, 23–2, Other (specify)

Another objective of our survey was to verify whether the establishment of a Dual Career regulation could increase the number of student-athletes enrolled at the University of Rome “Tor Vergata”, combining competitive sport activity with university studies. Therefore, we carried out a data collection from the registers of the University and the CUS, monitoring the number of student-athletes who activated the Dual Career after the introduction of the special regulation, in the academic years 2022/2023 and 2023/2024.

### 2.2 Statistical analysis

Statistical analysis was performed using GraphPad Prism 8 software (GraphPad Prism 8.0.1, La Jolla, CA, USA). Specifically, the collected responses were organized and coded for analysis by processing datasets for each of the key survey questions.

The distribution of responses was displayed with vertical bar graphs to show the total number of responses for each academic year. One-way ANOVA and Tukey's multiple comparison test were performed to compare the number of student respondents for each academic year who played competitive or recreational sport. Data were expressed as mean ± standard error and were considered significantly different if *p* < 0.05.

Contingency analysis was used to verify the existence of a significant distribution of competitive or recreational students across academic years, depending on the sport practised. Therefore, a contingency table was constructed to examine the joint distribution of the two categorical variables, applying the chi-square test to assess their association. The data were considered significantly different if *p* < 0.05.

Lastly, a pie chart was constructed to represent the total number of student-athletes for the academic years 2022/2023 and 2023/2024, with the relative percentages, in association with a vertical bar graph to show the distribution of the total number of enrolled student-athletes divided by each macroarea of the University of Rome “Tor Vergata”.

## 3 Results

**Figure 1:**
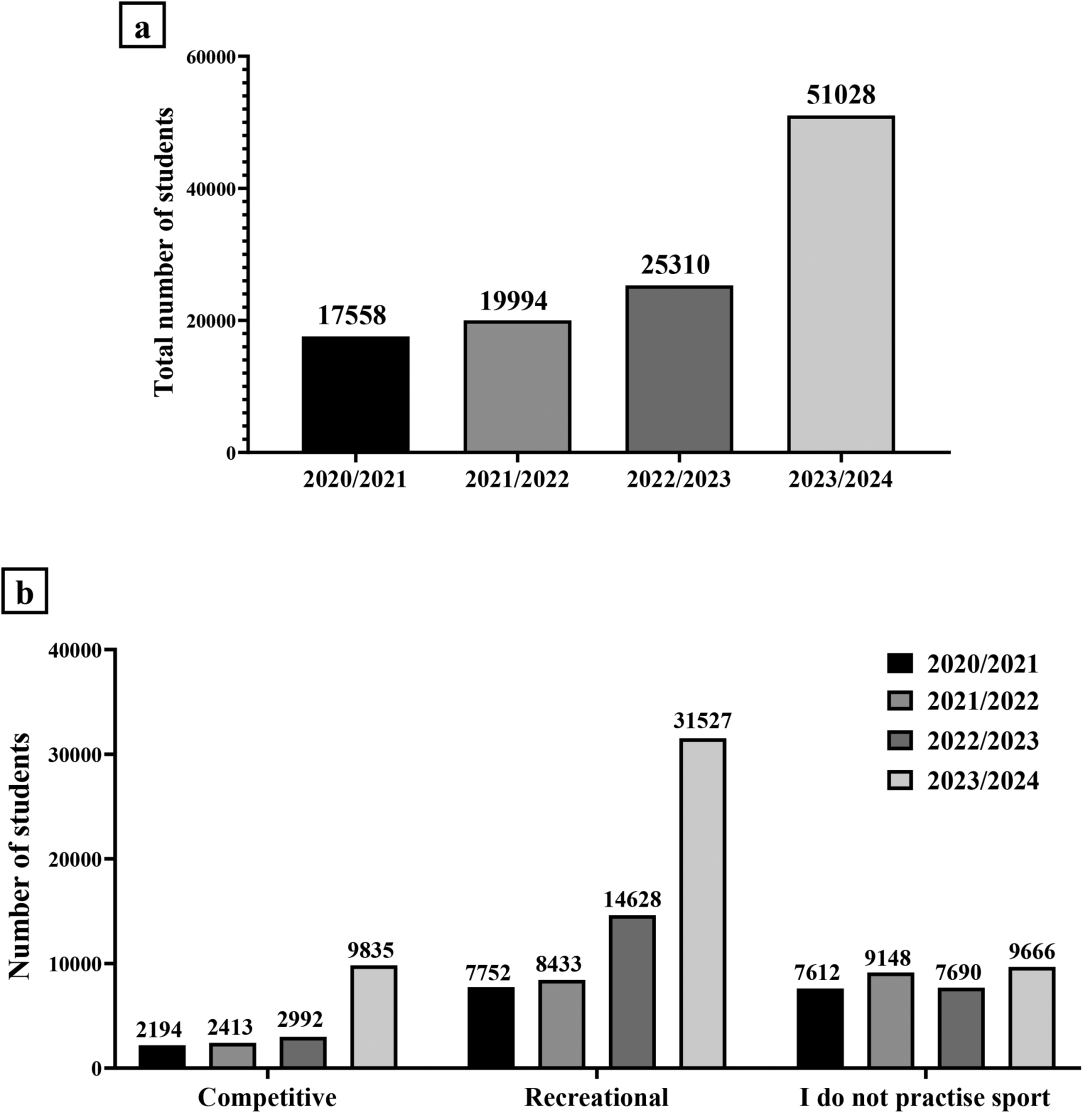
Distribution of students based on their participation in sports practice for each academic year. **(a)** The total number of students enrolled at the University of Rome “Tor Vergata” who participated in the survey was 17,558 in the academic year 2020/2021, 19,994 in the academic year 2021/2022, 25,310 in the academic year 2022/2023, and 51,028 in the academic year 2023/2024. **(b)** In the academic year 2020/2021, 2,194 students (13%) participated in competitive sports, 7,752 students (44%) participated in recreational sports and 7,612 students (43%) did not participate in sports. In the academic year 2021/2022, 2,413 students (12%) participated in competitive sports, 8,433 students (42%) participated in recreational sports and 9,148 students (46%) did not participate in sports. In the academic year 2022/2023, 2,992 students (12%) participated in competitive sports, 14,628 students (58%) participated in recreational sports and 7,690 students (30%) did not participate in sports. In the academic year 2023/2024, 9,835 students (19%) participated in competitive sports, 31,527 students (62%) participated in recreational sports and 9,666 students (19%) did not participate in sports.

Figure 1 illustrates the distribution of students according to their participation in sporting activities for each academic year.

Undoubtedly, an increasing number of students have been enrolled at the University of Rome “Tor Vergata” in the last four years, with the highest number recorded in the academic year 2023/2024 [Figure 1(a)]. Noteworthy, the percentage of students practising competitive or recreational sport increased in the academic years 2022/2023 and 2023/2024, in association with a reduction in the number of students not practising sport [Figure 1(b)]. Indeed, in the 2020/2021 academic year, 13% of students played competitive sport, 44% of students played recreational sport and 43% of students did not play sport. In the 2021/2022 academic year, 12% of students played competitive sport, 42% of students played recreational sport and 46% of students did not play sport. Although the number of students responding to the survey has increased over the academic years, in the 2022/2023 academic year, there was a clear increase in the number of students practising sport. Specifically, 12% of students played competitive sport, 58% of students played recreational sport and 30% of students did not play sport. In agreement, in the academic year 2023/2024, 19% of students played competitive sport, 62% of students played recreational sport and 19% of students did not play sport.

The comparison between the number of students participating in competitive and recreational sport for each academic year was performed using vertical bar graphs to visually compare data and detect trends over time.

**Figure 2:**
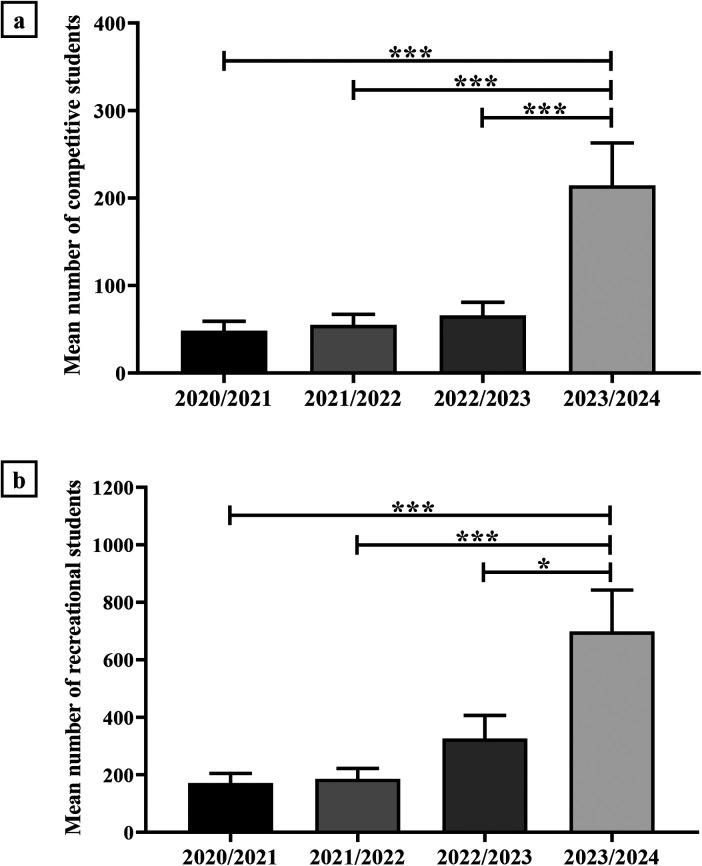
Comparison of the number of competitive and recreational students for each academic year. **(a)** Mean number of students participating in competitive sports: 48.3 ± 10.9 in the academic year 2020/2021, 54.9 ± 12.1 in the academic year 2021/2022, 65.8 ± 15.1 in the academic year 2022/2023, 214.4 ± 48.6 in the academic year 2023/2024 (***p < 0.001). **(b)** Mean number of students participating in recreational sports: 170.8 ± 33.9 in the academic year 2020/2021, 185.8 ± 36.4 in the academic year 2021/2022, 326.0 ± 80.7 in the academic year 2022/2023, 698.3 ± 144.7 in the academic year 2023/2024 (2020/2021 and 2021/2022 vs. 2023/2024, ***p < 0.001; 2022/2023 vs. 2023/2024, *p < 0.05).

Figure 2 shows a significant increase in the number of competitive and recreational sporting students in the 2023/2024 academic year compared to previous academic years. Indeed, the mean number of students participating in competitive sport was 48.3 ± 10.9 in the 2020/2021 academic year, 54.9 ± 12.1 in the 2021/2022 academic year, 65.8 ± 15.1 in the 2022/2023 academic year, and 214.4 ± 48.6 in the 2023/2024 academic year (***p < 0.001) [Figure 2(a)]. On the other hand, the mean number of students participating in recreational sports was 170.8 ± 33.9 in the 2020/2021 academic year, 185.8 ± 36.4 in the 2021/2022 academic year, 326.0 ± 80.7 in the 2022/2023 academic year, and 698.3 ± 144.7 in the 2023/2024 academic year (2020/2021 and 2021/2022 vs. 2023/2024, ***p < 0.001; 2022/2023 vs. 2023/2024, *p < 0.05) [Figure 2(b)].

A contingency analysis was performed to verify the existence of a significant distribution of competitive or recreational students across academic years, depending on the sport played.

**Figure 3:**
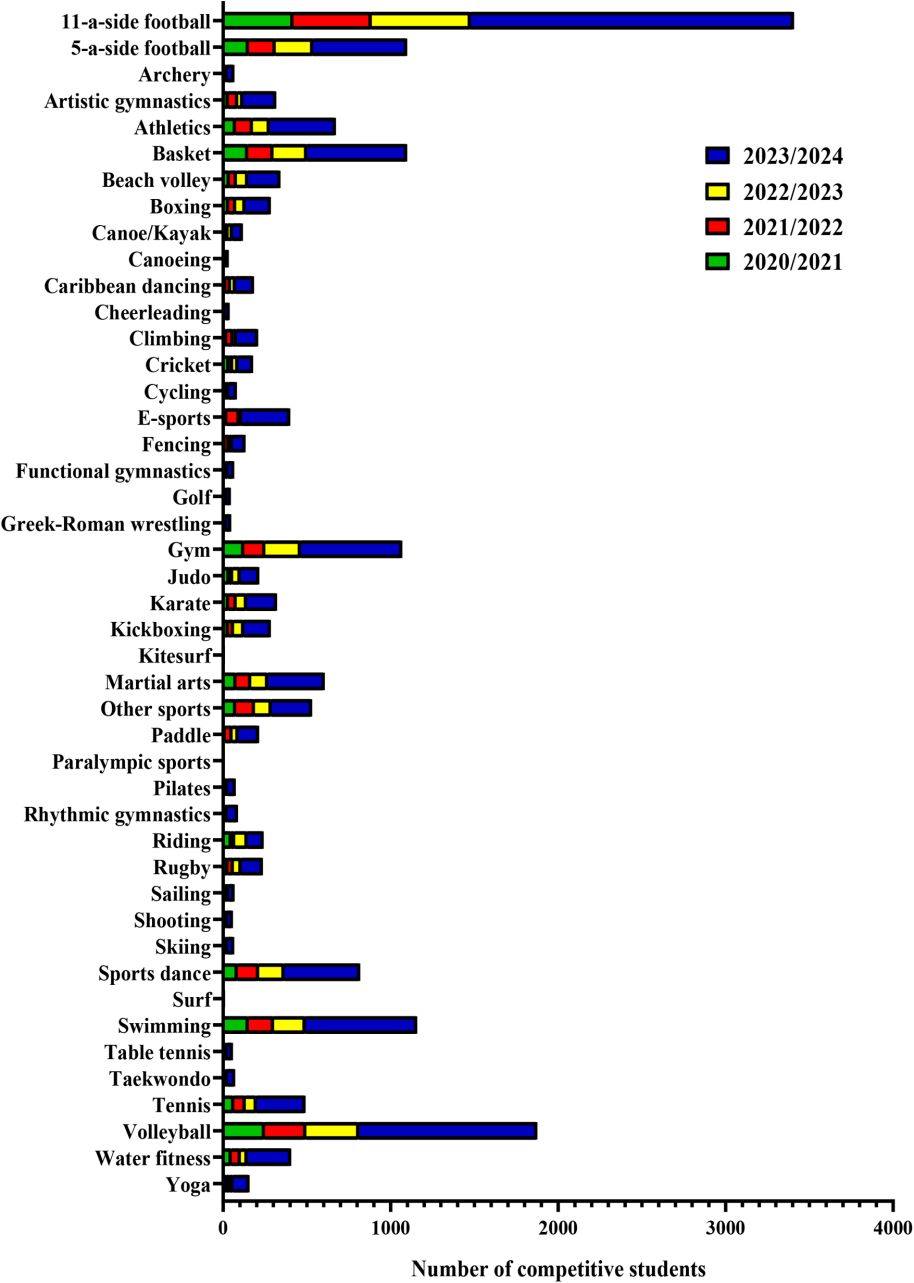
Distribution of competitive students by academic year and by sport using contingency analysis. The highest number of students participating in competitive sports was recorded for 11-a-side football, 5-a-side football, basket, gym, swimming and volleyball. Less marked variations or no variation in the number of competitive students across the four academic years were observed for other sports (*χ*² = 357, ****p < 0.0001).

Table 1 summarizes the distribution of competitive students for each academic year and each sport, while Figure 3 visually displays this data, allowing an immediate comparison between sports and years. Interestingly, the number of students playing 11-a-side football and 5-a-side football increased steadily over the four academic years. High student numbers were also recorded for basketball, gymnastics, swimming and volleyball, with higher participation in the 2022/2023 and 2023/2024 academic years. For all other sports, the number of competitive students remained the same or changed less markedly between the four academic years considered. Overall, the contingency analysis found that the differences in the number of students participating in competitive sports were statistically significant (*χ*² = 357, ****p < 0.0001), indicating that the observed increase was not due to chance.

**Table 1:** A schematic representation of the number of students participating in competitive sports for each academic year.

Sport	2020/2021	2021/2022	2022/2023	2023/2024
11-a-side football	419	468	592	1,929
5-a-side football	154	160	224	560
Archery	10	9	8	40
Artistic gymnastics	33	56	31	197
Athletics	76	101	102	394
Basket	149	151	202	597
Beach volley	40	42	67	193
Boxing	35	42	57	150
Canoe/Kayak	14	18	26	60
Canoeing	3	6	9	15
Caribbean dancing	19	28	29	108
Cheerleading	7	2	6	24
Climbing	24	37	21	127
Cricket	38	21	32	88
Cycling	7	16	12	47
E-sports	24	75	14	287
Fencing	14	25	19	76
Functional gymnastics	6	6	19	35
Golf	8	6	9	23
Greek-Roman wrestling	5	7	10	26
Gym	125	127	213	604
Judo	39	20	45	112
Karate	35	46	62	179
Kickboxing	33	32	60	159
Kitesurf	2	/	4	4
Martial arts	79	88	102	337
Other sports	77	113	100	241
Paddle	15	41	36	122
Paralympic sports	1	2	2	4
Pilates	12	9	9	45
Rhythmic gymnastics	7	13	8	60
Riding	51	21	74	95
Rugby	31	33	46	127
Sailing	13	8	15	32
Shooting	8	7	11	32
Skiing	8	9	12	37
Sports dance	87	127	152	452
Surf	1	1	1	8
Swimming	153	151	189	665
Table tennis	9	7	10	32
Taekwondo	10	7	9	45
Tennis	66	69	66	290
Volleyball	249	247	317	1,062
Water fitness	51	54	41	260
Yoga	24	18	19	96

**Figure 4:**
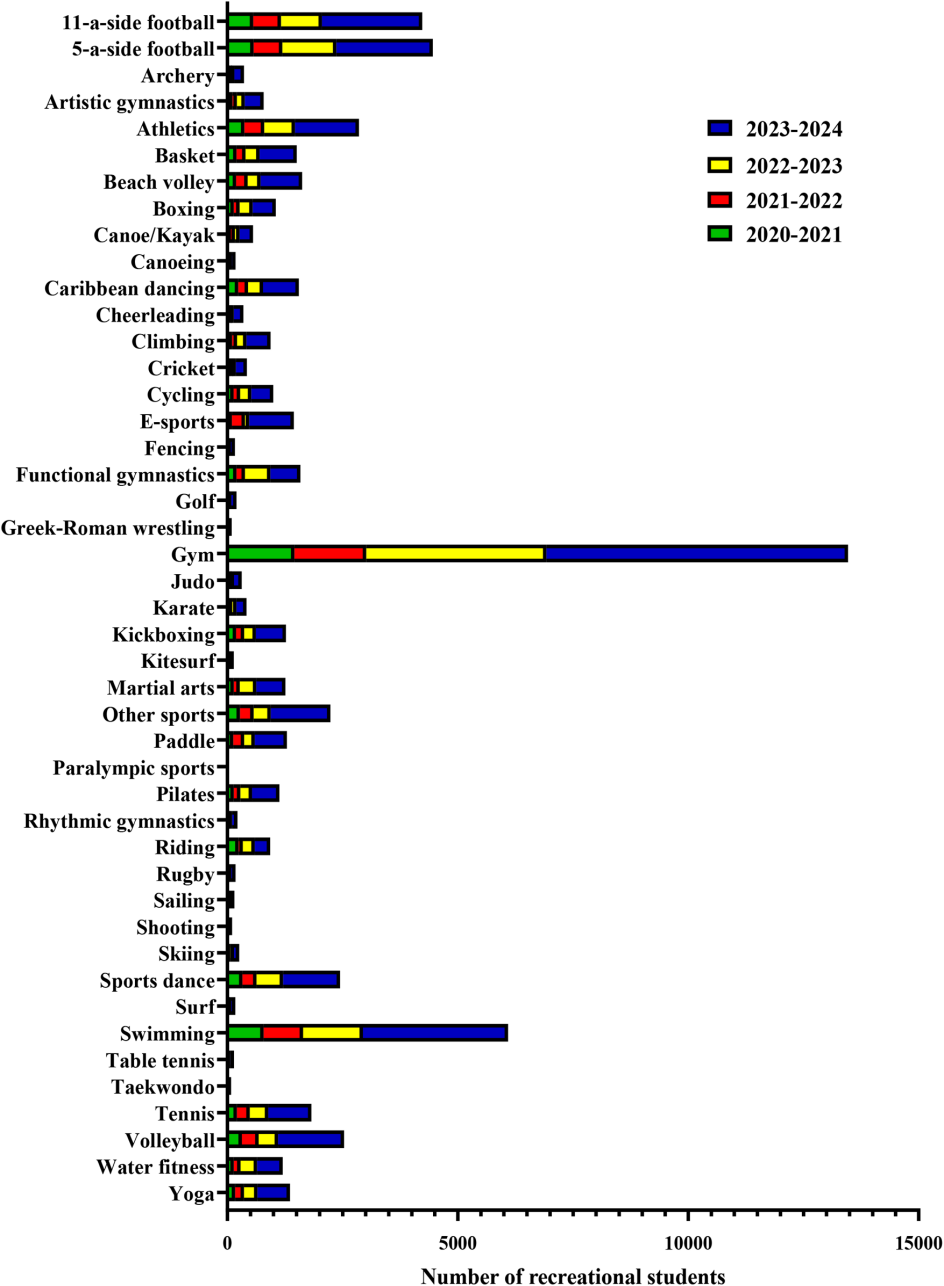
Distribution of ricreational students by academic year and by sport using contingency analysis. The highest number of students participating in competitive sports was recorded for gym, swimming, 11-a-side football and 5-a-side football. Less marked variations or no variation in the number of competitive students across the four academic years were observed for other sports (*χ*² = 1,495, ****p < 0.0001).

Table 2 summarizes the distribution of recreational students for each academic year and each sport, in addition to Figure 4 which visually illustrates this data and allows for immediate comparison between sports and years.

**Table 2:** A schematic representation of the number of students participating in recreational sports for each academic year.

Sport	2020/2021	2021/2022	2022/2023	2023/2024
11-a-side football	560	602	892	2,176
5-a-side football	571	622	1,174	2,092
Archery	43	61	50	209
Artistic gymnastics	109	98	172	409
Athletics	368	434	666	1,386
Basket	198	195	308	805
Beach volley	192	250	278	902
Boxing	141	125	285	500
Canoe/Kayak	78	87	98	295
Canoeing	25	37	36	80
Caribbean dancing	238	210	325	778
Cheerleading	44	58	39	207
Climbing	104	108	203	524
Cricket	55	69	61	237
Cycling	135	144	242	476
E-sports	97	286	93	966
Fencing	23	23	31	86
Functional gymnastics	197	187	550	650
Golf	28	28	29	113
Greek-Roman wrestling	16	11	17	48
Gym	1,454	1,560	3,910	6,544
Judo	36	53	55	167
Karate	43	64	93	217
Kickboxing	191	175	253	652
Kitesurf	22	23	24	72
Martial arts	138	130	360	633
Other sports	274	298	369	1,295
Paddle	127	239	231	697
Paralympic sports	2	2	/	6
Pilates	138	142	256	595
Rhythmic gymnastics	29	35	39	116
Riding	243	92	261	333
Rugby	28	25	33	91
Sailing	20	23	34	76
Shooting	16	17	16	53
Skiing	42	24	78	113
Sports dance	327	307	574	1,236
Surf	24	27	36	88
Swimming	788	854	1,300	3,150
Table tennis	18	17	30	78
Taekwondo	11	12	18	42
Tennis	208	278	402	936
Volleyball	317	360	423	1,432
Water fitness	138	148	358	556
Yoga	169	191	295	705

Contingency analysis shows that gym is the most popular recreational sport, with increasing participation per academic year. High numbers of students were also registered for swimming, while 11-a-side football and 5-a-side football were among the most popular recreational sports, with higher participation in the 2022/2023 and 2023/2024 academic years. About the other sports, less marked variations or no variations were observed in the number of recreational students between the four academic years considered. Overall, the increase observed with the contingency analysis is not due to chance, as statistically significant differences were found in the number of students playing recreational sports (*χ*² = 1495, ****p < 0.0001).

Another objective of our study was to examine the distribution of Dual Career student-athletes at the University of Rome “Tor Vergata”, after the approval of the appropriate regulation in April 2022. Therefore, our analysis focused on the distribution of student-athletes in the academic years 2022/2023 and 2023/2024 and the breakdown by macroarea of enrolment, evaluating the trend of Dual Careers in the last two academic years.

**Figure 5:**
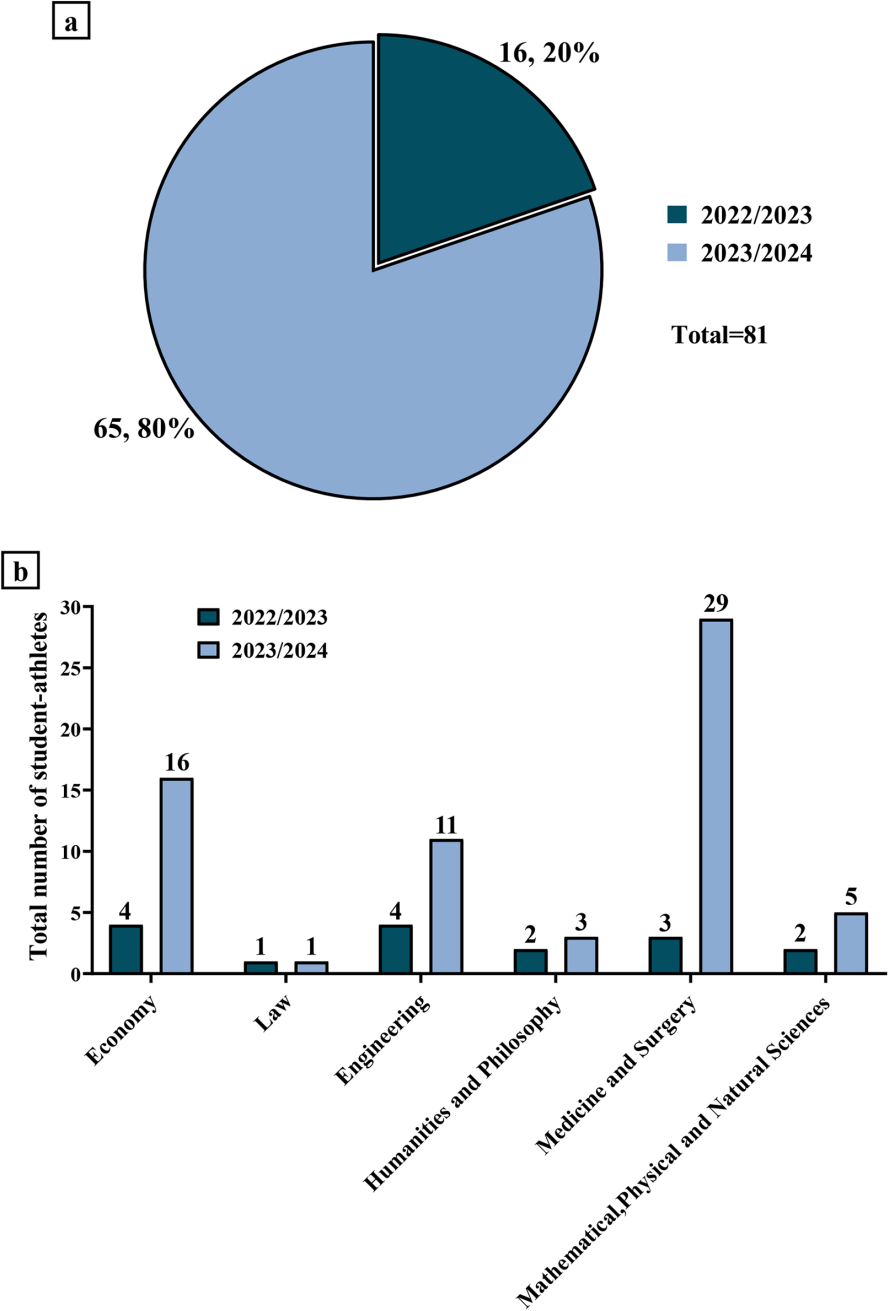
Distribution of student-athletes who activated the dual career program at the university of Rome “Tor Vergata” in the academic years 2022/2023 and 2023/2024. **(a)** 16 students (20%) activated the Dual Career program in the academic year 2022/2023, while 65 students (80%) activated the Dual Career program in the academic year 2023/2024. **(b)** Academic year 2022/2023: the number of student-athletes who activated the Dual Career program was 4 for the Economy macroarea, 1 for the Law macroarea, 4 for the Engineering macroarea, 2 for the Humanities and Philosophy macroarea, 3 for the Medicine and Surgery macroarea, and 2 for the Mathematical, Physical and Natural Sciences macroarea. Academic year 2023/2024: the number of student-athletes who activated the Dual Career program was 16 for the Economy macroarea, 1 for the Law macroarea, 11 for the Engineering macroarea, 3 for the Humanities and Philosophy macroarea, 29 for the Medicine and Surgery macroarea, and 5 for the Mathematical, Physical and Natural Sciences macroarea.

Specifically, Figure 5(a) depicts the number and relative percentage of student-athletes who activated Dual Careers in each of the last two academic years. Importantly, the percentage of students who took up Dual Careers increased from 20%–80% in the most recent academic year.

In addition, Figure 5(b) shows the distribution of student-athletes among the five macroareas of the University of Rome “Tor Vergata”, with marked variations between the two academic years considered. Unsurprisingly, the highest number of student-athletes with Dual Careers was recorded in the Medicine and Surgery macroarea, with an increase from 3–29, respectively, in the academic years 2022/2023 and 2023/2024. A similar trend was also observed in the Economy and Engineering macroareas. Regarding the Humanities and Philosophy and Mathematical, Physical and Natural Sciences macroareas, the number of student-athletes increased slightly in the most recent academic year, while no changes were found in the Law macroarea.

## 4 Discussion

Sport has a significant impact on the quality of life of individuals with psycho-physical and social effects, as it promotes the wellbeing of body and mind and encourages interaction and comparison between athletes (15). For this reason, Universities' monitoring of students engaged in sporting activities can provide relevant information and induce Universities to adopt strategies aimed at promoting and guaranteeing participation in sport during the university career. Therefore, our study aims to obtain an estimate of students involved in sport activities and to evaluate the effects of the introduction of a Dual Career regulation at the University of Rome “Tor Vergata”.

Our preliminary results show that the Dual Career regulation has been positively received, as the number of student-athletes enrolled in the University has increased in almost all macroareas since its introduction. Particularly, the marked increase in the number of student-athletes enrolled at the University of Rome “Tor Vergata” in the 2023/2024 academic year has shown that the facilities established by the regulations to protect athletes have promoted a greater enrolment in degree courses in the Medicine and Surgery and Economy macroareas. In addition, a significant increase in the number of students engaged in both competitive and recreational sporting activities was observed, at the same time as a reduction in the number of students not practising sport.

In this regard, the University of Rome “Tor Vergata” promotes numerous initiatives in sport and health aimed at both the prevention of pathologies and the adoption of a healthy lifestyle based on regular physical activity to encourage students to take up sport. Undoubtedly, a regulation to protect athletes engaged in degree programmes is essential for those who decide to embark on a Dual Career, as this requires a constant commitment and scrupulous organization of resources, including time and energy, ensuring the success of both paths (4). In fact, the success of a sports career is strictly dependent on the individual's technical characteristics, which are developed with high training volumes, especially in individual disciplines such as swimming, gymnastics and martial arts (14, 16). On the other hand, individual sports offer athletes the possibility of customizing training schedules according to the athlete's needs, while team sports impose constraints related to the needs of the community. In this context, several studies have examined Dual Career paths at various Universities, highlighting time management as the factor that most concerns student athletes. In fact, in addition to the time they must devote daily to training and academic life, the time needed to travel from one location to another must also be considered (17, 18). Therefore, the possibility of flexible working hours, guaranteed by the Dual Career regulation, makes it possible to overcome time management obstacles, favoring full and harmonious adherence to both careers.

In addition, athletes face significant financial burdens to support the university course, paying fees and books, and the sports course, which includes the costs of training, equipment and participation in competitions, including accommodation and meals (19). In this respect, the partial exoneration of university fees guaranteed by the regulation, in addition to reducing the economic pressure to which student-athletes are subjected, could promote an economic gain for the Universities through the visibility they produce. In fact, although most student-athletes do not enjoy the notoriety that allows them to generate income on their own, the involvement of followers through shares on social networks can increase the popularity of the University and encourage the dissemination of values related to sport (19, 20).

Finally, the social and relational impact that the Dual Career programme entails in the lives of individuals should be considered, as the difficulties associated with managing time, energy and financial resources often result in a sacrifice in interpersonal relationships on the part of the student-athlete. In this context, evidence has shown the importance of support from parents, siblings and coaches on a personal, sporting and university level to achieve success in all areas involved (8). Noteworthy, the period of social isolation experienced during the COVID-19 pandemic altered the balance between the athlete and the social context, reducing both the possibility of training in appropriate sports environments and support from teachers (21). In other words, the health crisis faced during the pandemic highlighted the delicate balance between sporting practice, quality of life and emotional state to which student-athletes are exposed, as well as the importance of a proactive social fabric to ensure continued engagement in both sport and study (22). In fact, the return to the daily routine in the post-lockdown period emphasized a real difficulty in resuming training and recovery cycles, as it was reported that elite athletes tended to spend more time resting and less time training than in the pre-lockdown period (23). Therefore, the life of the student-athlete is a constant physical, psychological and emotional challenge that requires *ad hoc* protection to ensure the individual's success.

Overall, our preliminary results of the survey administered to students enrolled in the academic years 2020/2021, 2021/2022, 2022/2023 and 2023/2024 showed that, following the introduction of an *ad hoc* Dual Career regulation, an increasing number of student-athletes and sports students enrolled at the University of Rome “Tor Vergata”. This result shows that the facilities provided by the regulation, which are necessary for the continuation of both paths, are indeed required by athletes embarking on a university career. Noteworthy, the regulation introduced in April 2022 seems to meet the needs of student-athletes, although further surveys will have to be conducted to assess their level of satisfaction. Nevertheless, preliminary results show that the main barriers encountered by athletes have been overcome by the Dual Career regulation, promoting higher enrolment in tertiary education courses.

In conclusion, the constant commitment of the University of Rome “Tor Vergata” in promoting and disseminating the importance of a lifestyle based on physical activity and the values associated with sports has had a positive impact on the student population, encouraging their participation in various sports disciplines. Further monitoring surveys will be necessary to verify the effect of the events and activities promoted by the University on student sports participation.

## 5 Limits of the study

The high number of students responding to the survey has resulted in a substantial amount of data that requires thorough and targeted analysis. Therefore, the present study presents preliminary results with some limitations that must be considered when interpreting the results. Firstly, no differentiated analysis was conducted based on the age and gender of the students, limiting our understanding of potential differences that could influence academic performance and participation in sports activities. For student-athletes, the specific sport practiced was not considered. In addition, the subdivision of sports according to type (individual, couple, team) requires a more in-depth and targeted investigation to provide an adequate stratification of the entire student population. Undoubtedly, these elements represent an area for future investigation for a more comprehensive understanding of the phenomena studied. Furthermore, the analysis of interactions between demographic, sports, and academic variables would offer a more integrated and detailed understanding of the factors that influence students’ experiences and performance.

## Data Availability

The original contributions presented in the study are included in the article/Supplementary Material, further inquiries can be directed to the corresponding author.
